# Correction to: Transcriptomics of manually isolated *Amborella trichopoda* egg apparatus cells

**DOI:** 10.1007/s00497-019-00367-8

**Published:** 2019-02-25

**Authors:** María Flores-Tornero, Sebastian Proost, Marek Mutwil, Charles P. Scutt, Thomas Dresselhaus, Stefanie Sprunck

**Affiliations:** 1 Cell Biology and Plant Biochemistry, Biochemie-Zentrum Regensburg, University of Regensburg, Universitätsstrasse 31, 93053 Regensburg, Germany; 2 Max-Planck Institute for Molecular Plant Physiology, Am Muehlenberg 1, 14476 Potsdam, Germany; 3 School of Biological Sciences, Nanyang Technological University, 60 Nanyang Drive, Singapore, 637551 Singapore; 4 Laboratoire Reproduction et Développement des Plantes, École Normale Supérieure de Lyon, Université Claude Bernard Lyon 1, CNRS, INRA, Université de Lyon, Lyon, France; 5 Present Address: Laboratory of Molecular Bacteriology (Rega Institute), KU Leuven, Louvain, Belgium

## Correction to: Plant Reproduction 10.1007/s00497-019-00361-0

The article Transcriptomics of manually isolated *Amborella trichopoda* egg apparatus cells, written by María Flores-Tornero, Sebastian Proost, Marek Mutwil, Charles P. Scutt, Thomas Dresselhaus, Stefanie Sprunck, was originally published electronically on the publisher’s internet portal (currently SpringerLink) on 1 February 2019 without open access. With the author(s)’ decision to opt for Open Choice, the copyright of the article changed on 22 February 2019 to © The Author(s) 2019 and the article is forthwith distributed under the terms of the Creative Commons Attribution 4.0 International License (http://creativecommons.org/licenses/by/4.0/), which permits use, duplication, adaptation, distribution and reproduction in any medium or format, as long as you give appropriate credit to the original author(s) and the source, provide a link to the Creative Commons license and indicate if changes were made.

The original article has been corrected.

